# Insights into protein kinase regulation and inhibition by large scale structural comparison

**DOI:** 10.1016/j.bbapap.2009.10.013

**Published:** 2010-03

**Authors:** Jeyanthy Eswaran, Stefan Knapp

**Affiliations:** a Structural Genomics Consortium, Nuffield Department of Medicine, University of Oxford Old Road Campus Building, Oxford OX3 7DQ, UK; b Department of Clinical Pharmacology, University of Oxford, Old Road Campus Building, Oxford OX3 7DQ, UK

**Keywords:** Protein kinase, Structure based drug design, X-ray crystallography, Atypical kinase, Kinase regulation

## Abstract

Protein structure determination of soluble globular protein domains has developed into an efficient routine technology which can now be applied to generate and analyze structures of entire human protein families. In the kinase area, several kinase families still lack comprehensive structural analysis. Nevertheless, Structural Genomics (SG) efforts contributed more than 40 kinase catalytic domain structures during the past 4 years providing a rich resource of information for large scale comparisons of kinase active sites. Moreover, many of the released structures are inhibitor complexes that offer chemical starting points for development of selective and potent inhibitors. Here we discuss the currently available structural data and strategies that can be utilized for the development of highly selective inhibitors.

## 1 Introduction

Protein phosphorylation is fundamental to all aspects of cell organization and behaviour. The interplay of 518 human kinases and the phosphatase family creates a signalling network that controls most signalling processes through reversible phosphorylation [1]. Malfunction of kinases has been identified as a common mechanism for many diseases including cancer, inflammation, infection and neurodegeneration [2]. Accordingly, kinase inhibitor development has been one of the major focuses of drug development during the past two decades [3–5], a considerable effort that led to the approval of 8 low molecular weight drugs so far. However, due to tremendous difficulties in developing highly selective inhibitors, most kinome targeted drug development programs restrict application to the oncology area [6]. Indeed, recent developments of screening platforms that cover most human kinases revealed that many currently used inhibitors inhibit several largely diverse kinases suggesting that cross reactivity of kinases is difficult to predict. Here, we discuss how the large-scale structural determination and small molecule screening efforts contribute towards understanding of phosphorylation dependent signalling in general and kinome targeted drug development.

**Fig. 1:**
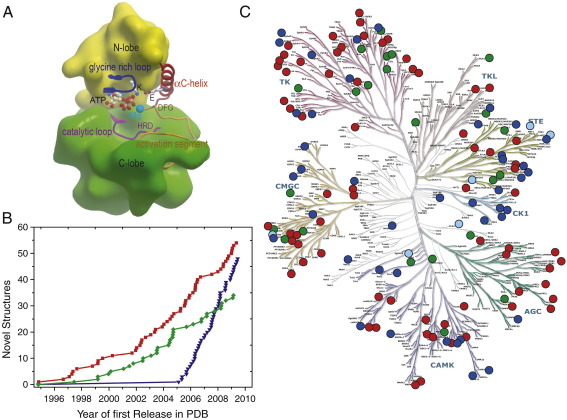
Kinase architecture and available structural knowledge. (A) Kinase domain organization and structural elements that regulate kinase activity. The upper and lower lobe of the kinase catalytic domain is highlighted in yellow and green, respectively. Elements important for catalytic activity are highlighted and labelled. (B) Release of new kinase structures into the protein data bank (http://www.rcsb.org/pdb/home/home.do) by academic (red), industrial (green) and structural genomics groups (blue). Shown are only human crystal structures considering the date when the structure has been released for the first time. (C) Available crystal structures of kinase catalytic domains mapped onto the phylogenetic tree of the human kinome [38].

Eukaryotic protein kinase domains (ePKs) possess highly conserved architecture comprising an N-terminal lobe with the conserved regulatory helix αC, and a larger mainly α-helical C-terminal lobe (Fig. 1A). The active site is located at the interface between the two kinase lobes and contains a number of highly conserved motifs that are essential for catalytic activity. Key catalytic motifs include the ATP/Mg^2+^ binding motifs “VIAK” and “DFG,” the catalytic HRD motif and the activation segment. A large number of kinases are activated by phosphorylation of the activation segment which is typically disordered in its inactive state and assumes a stable structure suitable for substrate binding in its phosphorylated active state [7–9].

## 2 The human kinome: current structural coverage

Currently there are 136 unique human catalytic domain structures (26% of all kinases) in the protein databank (PDB) (Fig. 1). However, the structural knowledge is not equally distributed and for many kinase groups there are only few structural models available [10]. In addition, a large fraction of kinases share less than 30% sequence homology with the closest related target of known structure. Moreover, the diversity of regulatory mechanisms that have been unravelled by structural studies makes this protein family still a very interesting target for structural investigations. The complex regulatory mechanisms of kinases that often differ even between closely related proteins are also the main reason why despite the large number of known kinase structures academic structural chemistry laboratories did not loose interest in kinases and continue to contribute about 8 novel kinase structures per year (Fig. 1B). Structural genomics efforts released 48 new human kinase structures so far and therefore contributed significantly to the available structural knowledge of this protein family. However, even with the current rate of structure determination it will still take more than 2 decades until we will have a comprehensive structural coverage of the kinase family. The progress in this area is monitored on the SGC website (http://www.sgc.ox.ac.uk/research/kinases/) and other resources [11].

## 3 Contribution of SG for our understanding of kinase regulation

Structural comparison is a powerful method for the identification of new mechanisms underlying enzymatic regulation. However, structural features may be influenced by the crystalline state of the protein. High throughput technologies offer the possibility to generate several structures of the same protein or closely related isozymes that differ in crystal contact regions adding confidence to the interpretation of structural data. For instance, regulation of kinase autophosphorylation by activation segment exchange between two adjacent catalytic domains was initially observed in the crystal structure of the kinase CHK2 [12]. Subsequent analysis of structures released by SG revealed that this mechanism being conserved quite widely in kinases that autophosphorylate at non-consensus sites located in the activation segment establishing this structural feature as a general mechanism of regulation [13,14]. Structures of different conformational states that are populated during catalysis can be generated using different inhibitors. Kinase inhibitors often recognize a certain conformation of a kinase and stabilize this state during crystallization. For the family of p21 activating kinases (PAKs) the combination of closely related isozymes and different inhibitors revealed the conformational states that are likely to be adopted during catalysis [15]. Comparison of different PAK crystal structures showed that helix αC, a key regulatory element of kinase function, extended its helical structure by one turn at the N-terminus. The observed structural changes led to the formation of interactions between conserved residues which structurally link the glycine rich loop, αC and the activation segment and anchor αC in an active conformation. Multiple crystal structures revealed also the role of the SH2 domain in maintaining the active state of the tyrosine kinase Fes [16]. SG efforts on unusual kinase that lie at the center of the kinome tree revealed new architectures of regulatory elements and identified a novel structural organization of activation segments in MPSK1 [17] and the atypical kinase haspin [18]. Also the first truly inactive pseudokinase, VRK3, has been released by SG efforts. Comparison with the closely related active isoform VRK2 showed that VRK3 stabilizes a pseudoactive conformation by mimicking an ATP bound state by acidic residues. The observed alterations in the kinase active site generated a structure with an inaccessible electronegative active site that is catalytically inert and can serve as a stable platform for the recruitment of interaction partners [19]. Thus, the structural studies led to a deeper understanding of kinase regulation and provided new templates for structure based design of selective inhibitors.

## 4 Strategies for the development of selective inhibitors

One of the most successful strategies for the development of selective inhibitors is targeting of diverse inactive conformations of kinases. These inactive states comprise the DFG-out state, in which the conserved phosphate binding motif “DFG” changes conformation opening a large allosteric binding pocket. This conformation is crucial for the tight binding of the first approved kinase inhibitor gleevec to inactive ABL but the binding mode was only recognized long after this drug had been developed [20], targeting the DFG out conformation (type II inhibitors) developed into key strategy for the design of selective kinase inhibitors. The inactive states of kinases are much more diverse than the well conserved active state of kinases [6,21]. However, targeting the DFG out conformation *per se* does not guaranty more favourable selectivity profiles and many unexpectedly cross reacting targets have been identified for type II inhibitors as well. Other strategies aim to target unique active site features, for instance small residues located in the hinge gatekeeper position that open an additional binding pocket. In addition, targeting allosteric binding sites that are located outside the conserved ATP binding pocket is an emerging strategy that has generated very selective inhibitors [22,23].

Highly potent and selective ligands can also be developed using organometallic inhibitors. Metal centres offer a large chemical diversity by their ability to coordinate a wide variety of ligand spheres. This property made it possible to design ligands that showed outstanding shape complementarity to kinase active sites and that bind with sub-nanomolar potency to the protein kinases PIM1 and GSK3beta [24,25].

**Fig. 2:**
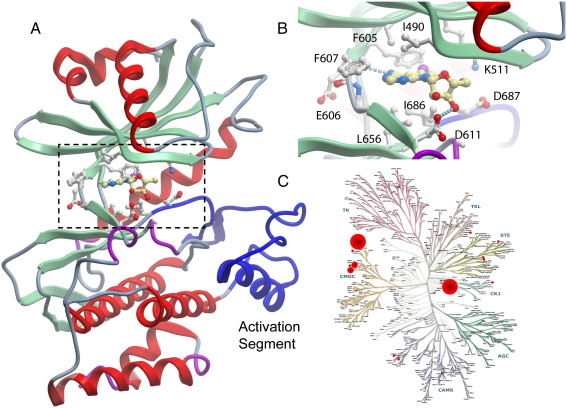
Binding of Iodotubercidine to haspin and selectivity of the inhibitor. (A) Structural overview showing the structure of the haspin kinase domain in complex with iodotubercidine (pdb: 2vuw). The atypical activation segment of the kinase this kinase is highlighted in blue. The active site shown in the detailed view in B is indicated by a dashed square. (B) Detailed view of the haspin active site in complex with iodotubercidine. Residues interacting with the inhibitor are labelled and shown in stick representation. (C) Selectivity profile showing hits in a screen of 10 μM iodotubercidine against a representative set of 98 human kinases. Hits that showed *T*_m_ shifts of more than 10 °C, representing low nm hits are highlighted by large red spheres, *T*_m_ shifts 10 °C > *T*_m_ > 6 °C (representing > 100 nM to μM hits) are shown by smaller spheres and hits 6 °C > *T*_m_ > 4 °C (μM hits) are indicated by very small spheres. Targets with *T*_m_ shifts smaller than 4 °C are not shown.

Finally, certain human ePKs have very diverse active sites and share less than 25% homology with other kinase family members. The diversity of the ATP site and the lack of certain motifs that are otherwise highly conserved in ePKs makes the design of selective inhibitors less challenging. A number of these diverse kinases have interesting links to disease. One examples for such an atypical kinase is haspin (haploid germ cell–specific nuclear protein kinase, encoded by Germ cell-specific gene 2; Gsg2) [26]. Haspin lacks both the conserved ATP/Mg^2+^ binding motif Asp-Phe-Gly (DFG), which is replaced by Asp-Tyr-Thr (DYT), and the Ala-Pro-Glu (APE) motif usually found at the C-terminus of the activation segment. In addition, haspin shares only weak sequence homology with ePKs and contains a highly divergent kinase domain with several unique inserts [27]. Haspin plays a key role during mitosis and is vital for the maintenance of chromosome cohesion. Depletion of haspin leads to a loss of cohesin association, premature chromatid separation and the failure of normal chromosome segregation [28–30]. Mitotic kinases have been recognized as appealing targets for the development of anti-mitotic drugs for cancer therapy. Although no direct link of haspin to cancer has been established so far this kinase plays an important role in the activation of the well-established oncology target Aurora B [31]. Inhibitors with reasonable selectivity profiles have been recently identified by screening of a targeted chemical library. In particular the inhibitor iodotubercidin cross-reacted only with a significant temperature shift with one other kinase (DYRK2) using a screening panel of 98 kinases using differential scanning calorimetry (Fig. 2). The high potency of iodotubercidin, its cell permeability and selectivity for haspin makes this compound an interesting tool to elucidate haspin function and it may serve as a good starting point for future inhibitor development.

A number of large cross screening panels have been developed recently that allow extensive profiling of kinase inhibitors [5,32–34]. However, the high cost of commercial selectivity screening usually precludes frequent use of the established panels for academic laboratories. Also only few large screening arrays are available to laboratories that are interested in the development of selective kinase inhibitors in academia [33,34]. Using the available reagents we started also to provide selectivity data for a large number of kinase targets using a differential scanning fluorimetry assay format [35]. This large body of structural and screening data can now be applied to understand mechanisms of inhibitor cross reactivity. Emerging strategies that could eliminate cross reactivity and enhance the specificity include out of the “Box” inhibitors (e.g. allosteric Inhibitors, regulatory domain and catalytic domain interfaces), utilising unique binding modes, unusual active site features and atypical kinases that attracted very little attention so far. In addition, non-human protein kinases expressed in pathogens as essential genes may offer an attractive strategy for treatment of infectious disease. These kinases are often structurally very diverse and may contain unique active site features that can be explored for selective inhibitor development [36,37]. Inhibitors that bind outside the largely conserved ATP binding pocket offer a promising strategy to improve selectivity. The allosteric site modulates kinase activity and exhibit the highest degree of kinase selectivity because they exploit binding sites and regulatory mechanisms that are unique to a particular kinase.

## 5 Conclusion

In recent years high throughput structure determination efforts provided a large resource of structural and chemogenomic information for the design of kinase inhibitors. Multiple structures determined in complex with different inhibitors locked kinase catalytic domain structures in a variety of different conformations. Comparison of these diverse conformational states provided insight into the dynamic features of kinases and unravelled new mechanisms of regulation.
